# Protective properties of geniposide against UV-B-induced photooxidative stress in human dermal fibroblasts

**DOI:** 10.1080/13880209.2018.1446029

**Published:** 2018-03-09

**Authors:** Daehyun Shin, Sihyeong Lee, Yu-Hua Huang, Hye-Won Lim, Yoonjin Lee, Kyounghee Jang, Yongwan Cho, Sang Jun Park, Dae-Duk Kim, Chang-Jin Lim

**Affiliations:** a R&D Center, Cosmocos Corporation, Incheon, Republic of Korea;; b College of Pharmacy and Research Institute of Pharmaceutical Sciences, Seoul National University, Seoul, Republic of Korea;; c Department of Biochemistry, Kangwon National University, Chuncheon, Republic of Korea;; d R&D Center, Shebah Biotech Inc, Chuncheon, Republic of Korea;; e KISSMYSKIN Co, Busan, Republic of Korea

**Keywords:** Anti-photoaging, glutathione, matrix metalloproteinase-2, Nrf2, reactive oxygen species, superoxide dismutase

## Abstract

**Context:** Geniposide (genipin-1-*O*-β-d-glucoside) is a major bioactive ingredient in the fruits of gardenia [*Gardenia jasminoides* J. Ellis (Rubiaceae)], a traditional herbal medicine in Asian countries.

**Objective:** This work assesses the skin anti-photoaging potential of geniposide in human dermal fibroblasts under UV-B irradiation.

**Materials and methods:** The anti-photoaging property of geniposide, at varying concentrations (5, 12 and 30 μM) treated for 30 min prior to UV-B irradiation, was evaluated by analysing reactive oxygen species (ROS), promatrix metalloproteinase-2 (proMMP-2), glutathione (GSH), superoxide dismutase (SOD), nuclear factor erythroid 2-related factor 2 (Nrf2) and cellular viability.

**Results:** Geniposide suppressed the ROS elevation under UV-B irradiation, which was revealed using three ROS-sensitive fluorescent dyes. The use of 2′,7′-dichlorodihydrofluorescein diacetate (DCFH-DA), dihydroethidium (DHE) and dihydrorhodamine 123 (DHR-123) elicited the IC_50_ values of 10.5, 9.8 and 21.0 μM, respectively. Geniposide attenuated proMMP-2 at activity and protein levels that were elevated under UV-B-irradiation. Geniposide at 5, 12 and 30 μM augmented the UV-B-reduced total GSH content to 1.9 ± 0.1-, 2.2 ± 0.2- and 4.1 ± 0.2-fold, respectively. Geniposide at 5, 12 and 30 μM upregulated total SOD activity to 2.3 ± 0.1-, 2.5 ± 0.3- and 3.3 ± 0.3-fold, respectively, under UV-B irradiation. The UV-B-reduced Nrf2 levels were also upregulated by geniposide treatment. Geniposide, at the concentrations used, was unable to interfere with cellular viabilities under UV-B irradiation.

**Discussion and conclusions:** After the skin anti-photoaging potential of geniposide may be further verified, it can be utilized as a safer resource in the manufacture of effective anti-aging cosmetics.

## Introduction

Ultraviolet (UV) radiation gives rise to harmful phenomena in human skin, which include tanning, sunburn, immune suppression, cancer, photoaging, etc. (Afnan et al. 2012). Photoaging is regarded as an extrinsic aging caused by environmental factors, including UV radiation, and basically characterized by wrinkles and dryness (Anandan et al. 2013). Among the three subtypes of UV radiation, UV-C radiation (wavelength, 100–280 nm) is almost completely absorbed by the ozone layer and has no harmful effect, UV-B radiation (280–315 nm) causes adverse effects on the superficial layer of the skin and is the most threatening environmental factor to photoaging, and UV-A radiation (315–400 nm) has a minor influence on the skin. Chronic exposure to UV-B radiation typically brings about the enhanced generation of reactive oxygen species (ROS) in the skin, which leads to oxidative stress and photodamages to macromolecules, such as proteins and nucleic acids (Jo et al. 2012). It also causes adverse changes in the extracellular matrix that lead to skin photoaging due to the enhanced production of matrix metalloproteinases (MMPs), including MMP-1, MMP-2 and MMP-9, which is possibly mediated by augmented ROS production (Ho et al. 2005).

**Figure 1.**
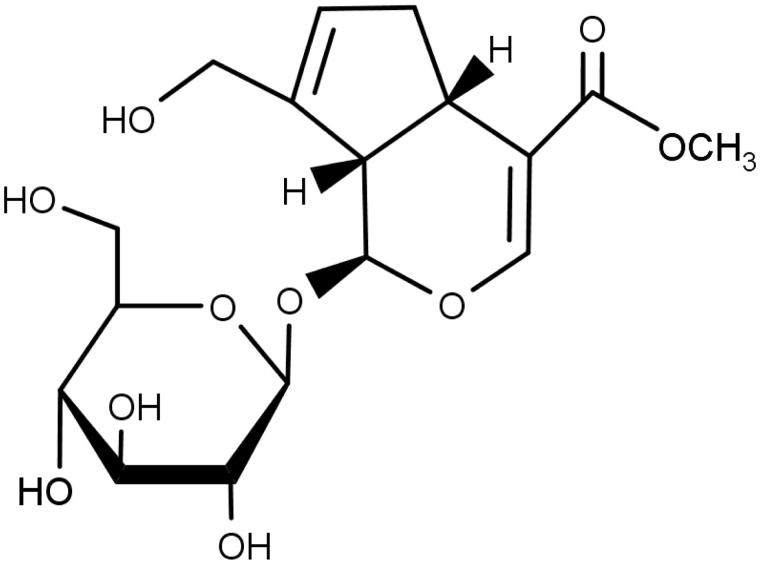
The chemical structure of geniposide.

The fruits of gardenia [*Gardenia jasminoides* J. Ellis, (Rubiaceae)] have been included in traditional formulations for the treatment of inflammation, jaundice, headache, oedema, fever, hepatic disorders and hypertension (Aburada et al. 1976; Tseng et al. 1995). Their main ingredients include iridoid glycosides, such as geniposide (Figure 1), gardenoide, genipin-1-*O*-β-gentiobioside, geniposidic acid, acetylgeniposide and gardoside (Bergonzi et al. 2012). Besides them, they contain crocin, crocin-3, crocetin, imperatorin, isoimperatorin, 5-hydroxy-7,3′,4′,5′-tetrainethoxyflavone and so on (Chen et al. 2007). Geniposide has been shown to have various biological and pharmacological activities, including neuroprotective (Chen et al. 2015), antidepressant (Cai et al. 2015), antinociceptive (Gong et al. 2014), anti-allergic (Sung et al. 2014), anti-angiogenic (Koo et al. 2004), anti-inflammatory (Chen et al. 2015), antidiabetic (Yao et al. 2015), antithrombotic (Suzuki et al. 2001) and hepatoprotective (Wang et al. 2015) activities.

At present, geniposide has been found to exert some of its known pharmacological effects through its antioxidant activity. It inhibits hypoxia/reoxygenation-induced myocardial apoptosis by reversing mitochondrial dysfunction through attenuating oxidative stress products, including ROS/reactive nitrogen species and malondialdehyde, and enhancing antioxidant enzymes (Jiang et al. 2016). Geniposide can protect cultured primary cortical neurons from amyloid-β-mediated mitochondrial dysfunction by reducing ROS production, recovering mitochondrial membrane potential and inhibiting apoptosis (Zhao et al. 2016). It diminishes amyloid-β-induced inflammation and oxidative stress by targeting receptor for advanced glycation end products (RAGE)-dependent signalling *in vivo* and *in vitro* (Lv et al. 2015). However, the beneficial effects of geniposide in human skin have not been clearly established. In the present work, the protective properties of geniposide against UV-B-induced photooxidative stress have been ascertained in human dermal fibroblasts, implying its potential use as a natural resource in the manufacture of anti-photoaging cosmetics.

## Materials and methods

### Chemicals

Geniposide (purity ≥98%), bovine serum albumin (BSA), Bradford’s reagent, 3-(4,5-dimethylthiazol-2-yl) 2,5-diphenyltetrazolium bromide (MTT), 2′,7′-dichlorodihydrofluorescein diacetate (DCFH-DA), dihydrorhodamine 123 (DHR-123), dihydroethidium (DHE), 5,5′-dithiobis(2-nitrobenzoic acid) (DTNB), glutathione (GSH), glutathione reductase (GR), catalase, xanthine, xanthine oxidase, cytochrome c and NADPH were purchased from Sigma-Aldrich Chemical Co. (St Louis, MO). Cell lysis buffer [25 mM Tris-phosphate (pH 7.8), 2 mM 1,2-diaminocyclohexane-*N*,*N*,*Nv*,*Nv*-tetraacetic acid, 2 mM dithiothreitol, 10% glycerol, 1% Triton X-100] was obtained from Promega Korea (Seoul, Korea). All other chemicals used were of the highest grade commercially available.

### Cell culture

A human dermal fibroblast cell line, CCD986Sk (ATCC, Manassas, VA), was grown in Dulbecco’s Modified Eagle’s Medium (DMEM) containing 10% heat-inactivated foetal bovine serum (FBS), 100 μg/mL streptomycin and 100 units/mL penicillin in a humidified atmosphere with 5% CO_2_ at 37 °C.

### UV-B irradiation

As a source of UV-B radiation, an UV lamp (peak, 312 nm; model VL-6M, Vilber Lourmat, Marine, France) was used along with a radiometer (model VLX-3W, Vilber Lourmat, Marine, France) and a sensor (bandwidth, 280–320 nm; model CX-312, Vilber Lourmat, Marine, France). Fibroblasts were irradiated with solar simulated UV-B radiation at the intensity of 70 mJ/cm^2^ which was chosen to induce photooxidative stress based on the preliminary work.

### Preparation of cellular lysate

After adherent cells were twice washed with phosphate-buffered saline and stored on ice for 5 min, they were collected using a cell scraper. After centrifugation at 5000×*g* for 10 min, the cell pellets were resuspended in cell lysis buffer and stored for 30 min on ice. Cellular lysate was taken after centrifugation at 5000×*g* for 15 min.

Protein contents in cellular lysates were quantitated according to the Bradford protein assay (Bradford 1976) using BSA as a reference protein.

### Quantitation of intracellular ROS

An ROS-sensitive probe DCFH-DA, which generates the fluorescent 2′,7′-dichlorofluorescein (DCF; *λ*_excitation_ = 485 nm, *λ*_emission_ = 530 nm) upon enzymatic reduction and subsequent oxidation by ROS, was used (Royall and Ischiropoulos 1993). Two other ROS probes, such as DHE and DHR-123, were also used in a similar manner, which produce fluorescent 2-hydroxyethidium (2-HE; *λ*_excitation_ = 480 nm, *λ*_emission_ = 525 nm) and rhodamine 123 (RH-123; *λ*_excitation_ = 500 nm, *λ*_emission_ = 535 nm), respectively, upon reaction with ROS. After cells were treated with geniposide and/or 20 μM DCFH-DA, 5 μM DHE or 5 μM DHR-123 for 30 min at 37 °C, they were twice washed with 1 mL FBS-free DMEM. The cells, resuspended in 1 mL FBS-free DMEM, were irradiated. The ROS levels were quantitated by monitoring fluorescence using multi-mode microplate reader (Synergy™ Mx, BioTek Instruments, Winooki, VT).

### MTT assay

Cell viabilities were determined using MTT assay based on metabolic activity (Freshney 1994). The amount of formazan, produced from reduction of MTT by the mitochondria of living cells, was quantitated by monitoring absorbance at 540 nm.

### Gelatin zymography

The gelatinolytic activity of promatrix metalloproteinase-2 (proMMP-2) in conditioned media was determined using zymographic analysis (Kleiner and Stetler-Stevenson 1994). After SDS-PAGE gel was stained with 0.1% Coomassie Brilliant Blue R-250, proMMP-2 activities were convinced as clear zones against a blue background. The proMMP-2 band was confirmed in accordance with its molecular mass, 72 kDa, which was identified by protein molecular weight markers.

### Western blotting analysis

To quantitate proMMP-2 and nuclear factor erythroid 2-related factor 2 (Nrf2) in cellular lysates, western blotting analysis was performed using anti-MMP-2 (ALX-210-753, Enzo Life Sciences, Farmingdale, NY) and anti-Nrf2 (ab31163, Abcam, Cambridge, MA) antibodies as primary antibodies. GAPDH, used as an internal loading control, was detected using anti-GAPDH antibody (LF-PA0212, AbFrontier, Seoul, Korea). In brief, cellular lysates were separated on 10% (w/v) SDS-PAGE and electrotransferred to PVDF membranes. The blotted membrane was blocked with blocking buffer (2% BSA in 1× TBS-Tween 20), probed with primary antibodies overnight at 4 °C, incubated with secondary antibody (goat anti-rabbit IgG-pAb-HRP-conjugate; ADI-SAB-300, Enzo Life Sciences, Farmingdale, NY) for 1 h at room temperature, and developed using an enhanced West-save up^TM^ (AbFrontier, Seoul, Korea).

### Quantitation of total GSH

Total GSH in cellular lysates was quantitated using an enzymatic recycling assay based upon GR (Nakagawa et al. 1990). The reaction mixture (200 μL), which contained 175 mM KH_2_PO_4_, 6.3 mM EDTA, 0.21 mM NADPH, 0.6 mM DTNB, 0.5 units/mL GR and cellular lysate, was incubated at 25 °C. Absorbance at 412 nm was monitored using a microplate reader. Total GSH content was reported as μg/mg protein.

### Determination of SOD activity

Total superoxide dismutase (SOD) activity in cellular lysates was determined as the reduction of cytochrome c with xanthine/xanthine oxidase system (Lee et al. 2002). The reaction mixture (200 μL) contained 50 mM phosphate buffer (pH 7.4), 0.01 units/mL xanthine oxidase, 0.1 mM EDTA, 1 μM catalase, 0.05 mM xanthine, 20 μM cytochrome c and cellular lysate. A change in absorbance at 550 nm was monitored using a microplate reader.

### Statistical analysis

The results were represented as mean ± SD. Differences between experimental groups were analysed using one-way ANOVA followed by *post hoc* Tukey’s HSD test for multiple comparisons. A *p* value <0.05 was considered statistically significant.

## Results

### Suppression of UV-B-induced ROS elevation

**Figure 2.**
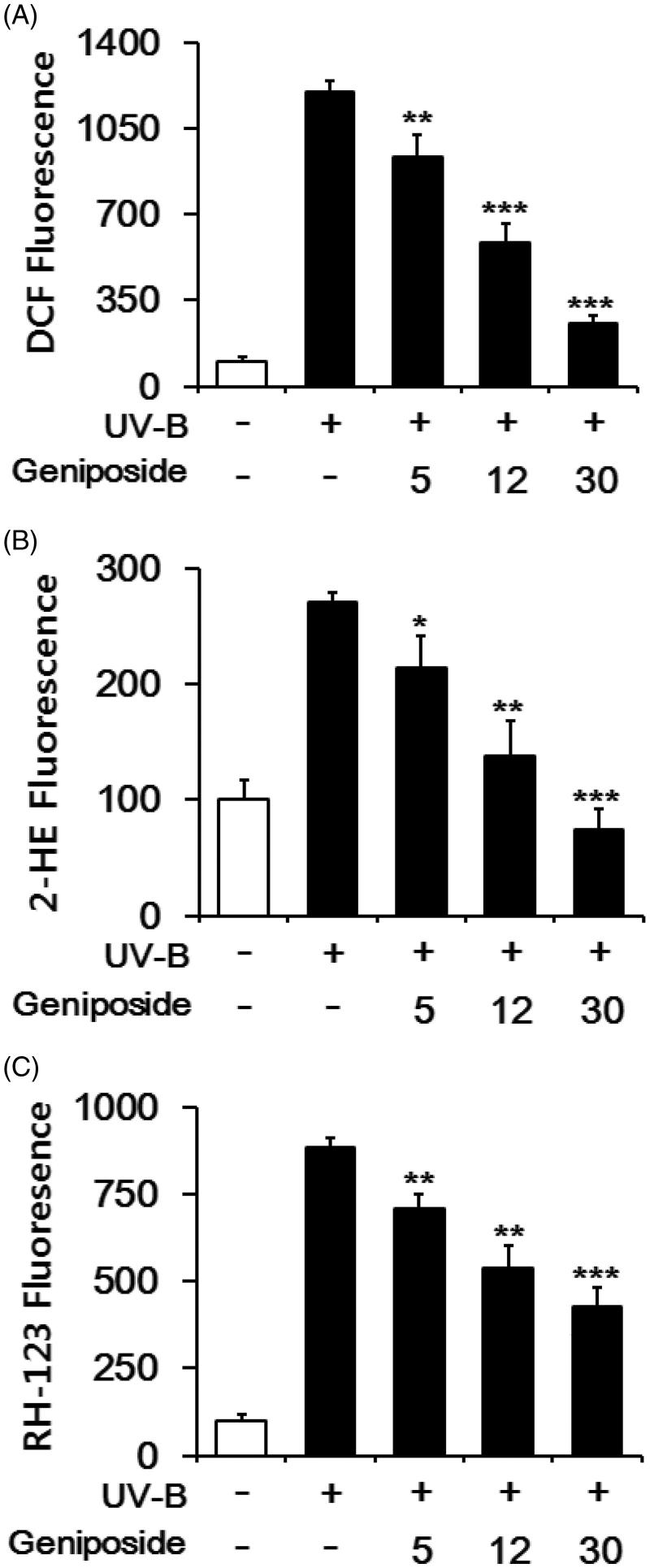
Attenuating effects of geniposide on the reactive oxygen species (ROS) elevation in human dermal fibroblasts under UV-B irradiation. Fibroblasts were subjected to the varying concentrations (0, 5, 12 or 30 μM) of geniposide for 30 min before the irradiation. The intracellular ROS levels were determined using DCFH-DA (A), DHE (B) and DHR-123 (C) in a microplate fluorometer. The intracellular ROS level was represented as DCF (A), 2-hydroethidium (2-HE, B) and rhodamine 123 (RH-123, C) fluorescence, expressed as % of the non-irradiated control. *p < 0.05; **p < 0.01; ***p < 0.001 versus the non-treated control (UV-B irradiation alone).

Fibroblasts were subjected to varying concentrations (0, 5, 12 or 30 μM) of geniposide prior to UV-B irradiation. When DCFH-DA was used as an ROS probe, the UV-B irradiation alone gave rise to about 12.3 ± 0.4-fold elevation in the ROS level over that in the non-irradiated control (Figure 2(A)). Geniposide at 5, 12 and 30 μM made the UV-B-induced ROS elevation reduce to 78.4 ± 6.8, 48.6 ± 6.0 and 21.6 ± 2.6% of the UV-B irradiation alone, respectively (Figure 2(A)). When DHE was used, the UV-B irradiation alone induced the ROS level to about 2.7 ± 0.1-fold over the non-irradiated control, and geniposide concentration-dependently attenuated the UV-B-induced ROS elevation (Figure 2(B)). When DHR-123 was used, geniposide similarly displayed an attenuation on the UV-B-induced ROS (Figure 2(C)). The use of DCFH-DA, DHE and DHR-123 gave rise to the IC_50_ values of 10.5, 9.8 and 21.0 μM, respectively. Collectively, geniposide suppresses the UV-B-induced ROS elevation in fibroblasts.

### Non-cytotoxicity

**Figure 3.**
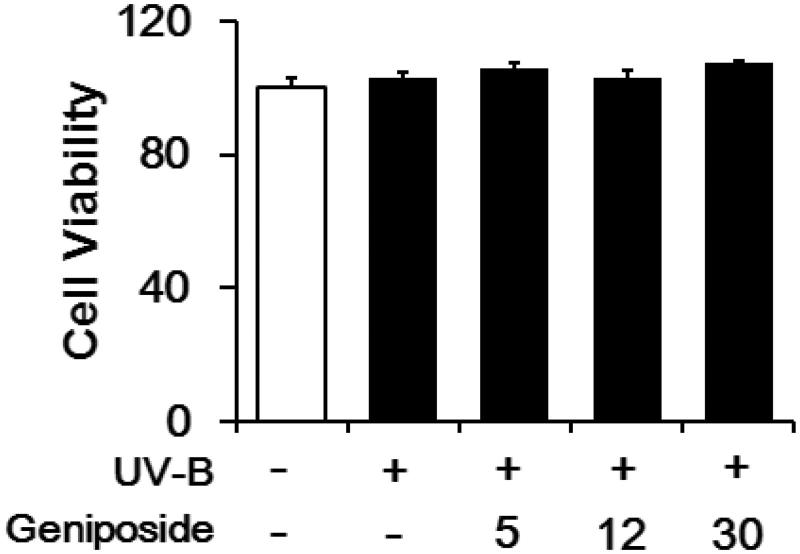
Effects of geniposide on cellular viability in human dermal fibroblasts under UV-B irradiation. The viable cell numbers, represented as % of the non-irradiated control, were determined using the MTT assay.

UV-B irradiation, under the experimental conditions used, was nontoxic to the viabilities of fibroblasts (Figure 3). Geniposide was unable to interfere with the viability of fibroblasts under UV-B irradiation (Figure 3).

### Attenuation of UV-B-induced proMMP-2 elevation

**Figure 4.**
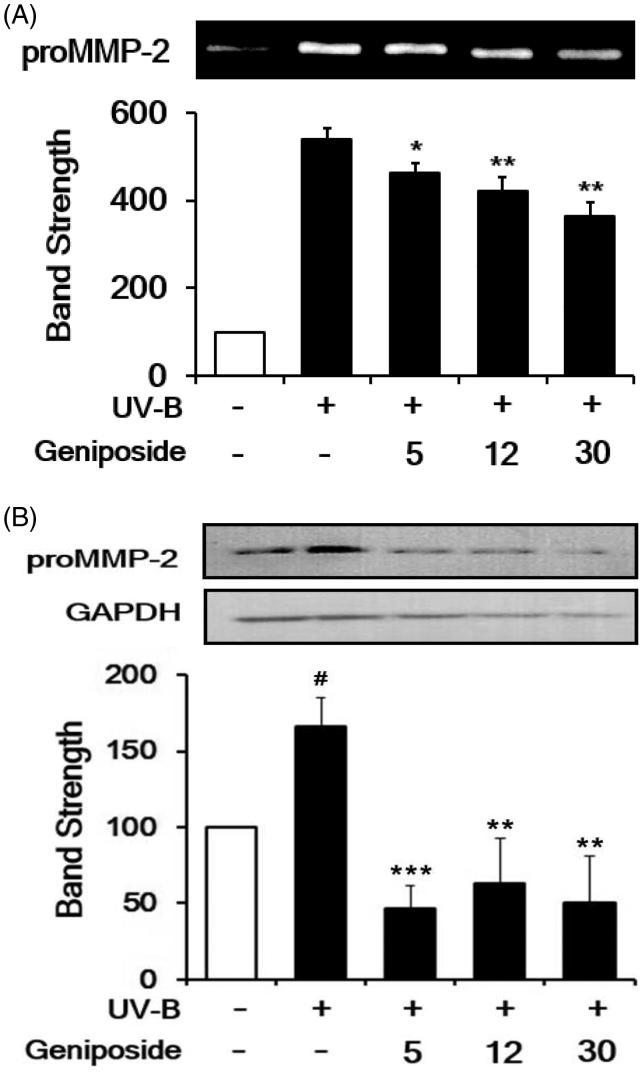
Attenuating effect of geniposide on the elevation of promatrix metalloproteinase-2 (proMMP-2) activity (A) and protein (B) levels in human dermal fibroblasts under UV-B irradiation. Fibroblasts were subjected to the varying concentrations (0, 5, 12 or 30 μM) of geniposide for 30 min before the irradiation. (A) The proMMP-2 gelatinolytic activity in conditioned medium was detected using gelatin zymography. (B) The proMMP-2 proteins in cellular lysates were determined using western blotting analysis with anti-MMP-2 antibodies. GAPDH was used as an internal loading control. A representative of the three independent results was shown. The band strength, represented as % of the non-irradiated control, was determined by densitometry using the ImageJ (version 1.48) software which is downloaded from the NIH website. (B) It was normalized to the corresponding GAPDH band. #*p* < 0.05 versus the non-irradiated control. (UV-B irradiation alone). *p < 0.05; **p < 0.01; ***p < 0.001.

As previously described (Kim et al. 2013), the UV-B irradiation enhanced the proMMP-2 gelatinolytic activity in cellular lysates to 5.5 ± 0.2-fold, compared with the non-irradiated control (Figure 4(A)). Geniposide at 5, 12 and 30 μM diminished the UV-B-induced proMMP-2 activity to 87.9 ± 3.8, 78.8 ± 5.3 and 69.7 ± 5.7% of the non-treated control, respectively (Figure 4(A)).

The UV-B irradiation alone significantly enhanced the proMMP-2 protein levels, compared to the non-irradiated control, and geniposide at 5, 12 and 30 μM markedly attenuated the UV-B-induced proMMP-2 protein levels (Figure 4(B)). In brief, geniposide down-regulates the UV-B-induced proMMP-2 at the protein level.

### Enhancement of UV-B-reduced total GSH

**Figure 5.**
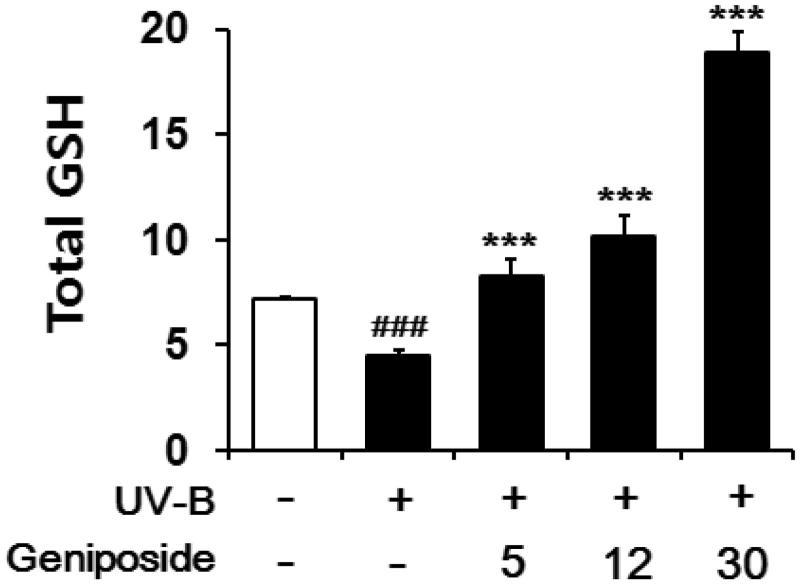
Enhancing effects of geniposide on total glutathione (GSH) levels in cellular lysates of human dermal fibroblasts under the irradiation with UV-B radiation. Fibroblasts were subjected to the varying concentrations (0, 5, 12 or 30 μM) of geniposide for 30 min before the irradiation. Total GSH content, expressed as μg/mg protein, was determined with enzymatic recycling assay using GR. ###*p* < 0.001 versus the non-irradiated control. ***p < 0.001 versus the non-treated control (UV-B irradiation alone).

In the irradiated fibroblasts, total GSH contents in cellular lysates were diminished to 64.4 ± 3.1% of the non-irradiated control (Figure 5), which was consistent with the previous finding (Zhu and Bowden 2004). Geniposide at 5, 12 and 30 μM enhanced the total GSH levels by 1.9 ± 0.1-, 2.2 ± 0.2- and 4.1 ± 0.2-fold, respectively, compared to the irradiated fibroblasts that was not pretreated with geniposide (Figure 5). Briefly, geniposide is capable of enhancing the UV-B-reduced total GSH levels in fibroblasts.

### Enhancement of UV-B-reduced SOD activity

**Figure 6.**
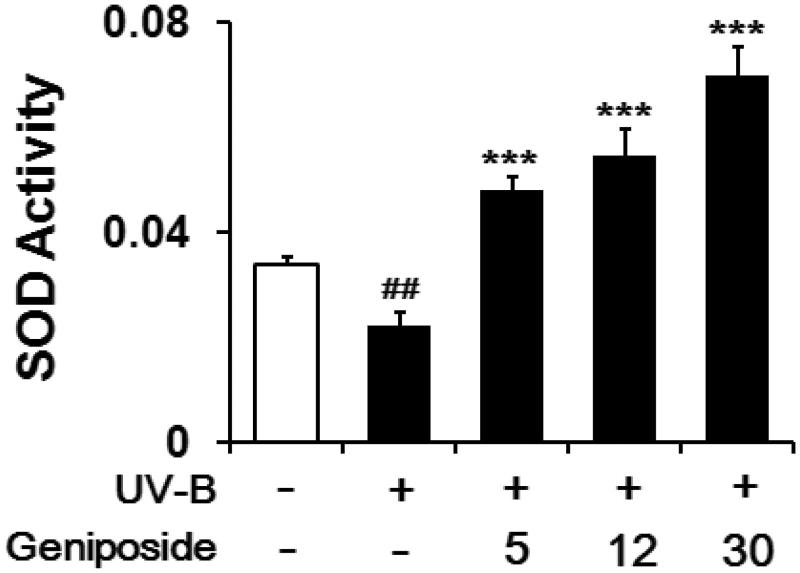
Enhancing effects of geniposide on total superoxide dismutase (SOD) activity levels in cellular lysates of human dermal fibroblasts under the irradiation with UV-B radiation. Fibroblasts were subjected to the varying concentrations (0, 5, 12 or 30 μM) of geniposide for 30 min before the irradiation. Total SOD activity, expressed as Δ_550_/min/mg protein, was measured using a spectrophotometric assay. ##*p* < 0.01 versus the non-irradiated control. ***p < 0.001 versus the non-treated control (UV-B irradiation alone).

When fibroblasts were irradiated with UV-B light, total SOD activity in cellular lysates was diminished to 64.8 ± 7.4% of the non-irradiated control (Figure 6). Geniposide at 5, 12 and 30 μM enhanced total SOD activity by 2.3 ± 0.1-, 2.5 ± 0.3- and 3.3 ± 0.3-fold, respectively, compared to the UV-B-irradiated fibroblasts without geniposide pretreatment (Figure 6). Briefly, geniposide enhances the UV-B-reduced total SOD activity in fibroblasts.

### Enhancement of UV-B-reduced Nrf2 levels

**Figure 7.**
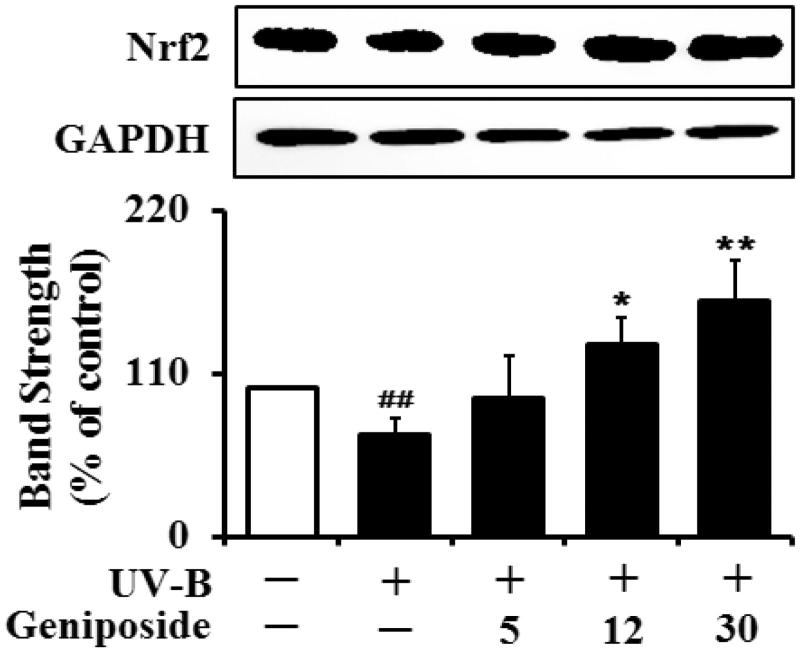
Enhancing effect of geniposide on the Nrf2 levels in human dermal fibroblasts under UV-B irradiation. Fibroblasts were subjected to fresh media with the varying concentrations (0, 5, 12 or 30 μM) of geniposide for 30 min before the irradiation. The Nrf2 proteins were determined using western blotting analysis with anti-Nrf2 antibodies. GAPDH was used as a protein loading control. The relative band strength was determined with densitometry using ImageJ software. Data are presented as % of control versus the non-irradiated control. ##*p* < 0.01 versus the non-irradiated control; *p < 0.05; **p < 0.01 versus the non-treated control (UV-B irradiation alone).

The Nrf2 levels in the UV-B-irradiated cells were dropped to 71.4 ± 9.3% of the non-irradiated control (Figure 7). Geniposide at 5, 12 and 30 μM could cause an enhancement in the UV-B-reduced Nrf2 levels by 1.3 ± 0.4-, 1.8 ± 0.3- and 2.3 ± 0.4-fold, respectively, compared to the UV-B irradiation only (Figure 7). Taken together, geniposide has an up-regulating activity on the UV-B-reduced Nrf2 levels in fibroblasts.

## Discussion

Gardenia fruits have been used for the treatment of diverse disorders, including inflammation, oedema and dermatitis, in traditional folk medicine. Geniposide, one of the main ingredients known to have both medicinal and nutritional value in gardenia fruits, has been assessed to be responsible for their diverse pharmacological actions. Geniposide protects against acute alcohol-induced liver injury through up-regulating the expression of the main antioxidant components, such as GSH, glutathione-*S*-transferase, GSH peroxidase, SOD and catalase, which thus ameliorates the alcohol-induced oxidative stress injury in the liver (Wang et al. 2015). It also enhances the levels of GSH and antioxidant enzymes, such as SOD and catalase, which are diminished in CCl_4_-treated mouse livers (Chen et al. 2016). Geniposide protects against the liver injury induced by tripterygium glycosides, commonly used as a basic medicine in the treatment of rheumatoid arthritis but with a high incidence of liver injury, in mice through the attenuation of oxidative stress and inflammation (Wang et al. 2016). It plays a protective role against lipopolysaccharide-induced acute lung injury by diminishing inflammatory responses via the activation of blocking nuclear factor-κB and mitogen-activated protein kinase signalling pathways (Xiaofeng et al. 2012). Geniposide suppresses the release of histamine from mast cells, which implies an anti-allergic effect and subsequently a therapeutic potential for atopic dermatitis (Sung et al. 2014). Penta-acetyl geniposide, an acetylated geniposide known to have hepatoprotective, anti-proliferative and apoptotic properties, exhibits an antimetastatic action in C6 glioma cells by mitigating MMP-2 levels (Huang et al. 2009). In the present work, we demonstrate that geniposide possesses radioprotective properties against UV-B irradiation by attenuating UV-B-induced ROS and proMMP-2 elevation, which proposes its skin anti-photoaging property. Our work further supports that the skin anti-photoaging property of geniposide might be based upon its up-regulating activity on antioxidant components, including total GSH and SOD activity tested in this work. This finding might correspond with the up-regulation of antioxidant components by geniposide in its hepatoprotective activity against the alcohol-induced oxidative stress injury in the liver (Wang et al. 2015). A recent report demonstrates that geniposide ameliorates 2,4,6-trinitrobenzene sulfonic acid-induced experimental rat colitis by reducing inflammation and modulating the disrupted epithelial barrier function via activating the AMP-activated protein kinase signalling pathway (Xu et al. 2017).

Diverse environmental factors, such as solar UV radiation and other external aggressors, can elicit an oxidative challenge that is detrimental to skin health. For example, continuing exposure to solar UV radiation leads to immune suppression, inflammation, photoaging and skin photocarcinogenesis. Since the levels of endogenous antioxidants decrease with age, thus resulting in a less protection and a greater potential for skin damage (Rodriguez et al. 2013), the improved strategies for skin photoprotection are needed, especially for elderly people. Pterostilbene, a natural phytoalexin, prevents an acute UV-B-induced increase in skin fold, thickness and redness, as well as photoaging-associated skin winkling and hyperplasia (Sirerol et al. 2015). It also prevents the chronic UV-B irradiation-induced skin carcinogenesis, which is associated with the maintenance of skin antioxidant defences, including GSH, catalase, SOD and GSH peroxidase, and the inhibition of UV-B-induced oxidative damage (Sirerol et al. 2015). These previous findings strongly suggest that certain compounds, either synthetic or natural, which are able to up-regulate the levels of antioxidant components, can act as effective skin anti-photoaging agents. Geniposide is been thought to be one candidate of such compounds, which can up-regulate at least two main antioxidant components, such as GSH and SOD.

Nrf2 is known as a redox-sensitive transcription factor which plays an important role in the protective process against UV-B-induced photoaging, originally based upon the fact that UV-B-irradiated *nrf2^–/–^* mice exhibit accelerated photoaging symptoms and decreased cutaneous GSH levels (Hirota et al. 2011). As a master regulator of the cellular antioxidant defence against environmental electrophilic insult, Nrf2 has emerged as a crucial determinant of cutaneous damage from solar UV, and the concept of pharmacological activation of Nrf2 has attracted considerable attention as a valuable approach to skin photoprotection (Tao et al. 2013). Nrf2 plays a protective role against UV-induced apoptosis *in vitro* and acute sunburn reactions *in vivo*, and prevents photoaging by preserving high levels of antioxidants, such as GSH, in the skin (Kim et al. 2015). In a human skin reconstruct exposed to solar simulated UV radiation, dihydrotanshinone, a phenanthrenequinone-based Nrf2 inducer, was found to cause an enhancement in Nrf2 and γ-glutamylcysteine synthetase levels together with the elevation of total GSH levels, and subsequently attenuate the occurrence of epidermal solar insult-markers, such as cleaved procaspase-3, pyknotic nuclei, eosinophilic cytoplasm and acellular cavities (Tao et al. 2013). Sulforaphane, an isothiocyanate derived from broccoli, induces the endogenous cellular defences regulated by Nrf2, including cytoprotective enzymes and GSH (Benedict et al. 2012). The apocarotenoid bixin, a natural food additive consumed worldwide, was previously shown to protect skin against solar UV-induced damage through the activation of Nrf2 (Tao et al. 2015). Geniposide is capable of up-regulating Nrf2 levels diminished under UV-B irradiation, suggesting that geniposide acts as an Nrf2 activator. Subsequently, geniposide causes enhancements in antioxidant components, which may result from its up-regulation of Nrf2. Currently, geniposide is assumed to have its defensive properties against photooxidative stress via the mediation of Nrf2.

## Conclusions

Geniposide suppresses the UV-B-induced elevation of ROS and proMMP-2 in human dermal fibroblasts. On the contrary, it augments the UV-B-reduced total GSH and SOD activity levels under UV-B irradiation. Geniposide up-regulates the UV-B-reduced Nrf2, a major transcriptional regulator that mediates the expression of a variety of endogenous antioxidants and detoxifying genes. Collectively, geniposide plays an anti-photoaging role in human skin under UV-B irradiation, possibly via up-regulation of Nrf2. This finding implies geniposide and geniposide-containing mixtures can be utilized as a natural resource in the manufacture of anti-photoaging cosmetics.
